# Radiomics based likelihood functions for cancer diagnosis

**DOI:** 10.1038/s41598-019-45053-x

**Published:** 2019-07-01

**Authors:** Hina Shakir, Yiming Deng, Haroon Rasheed, Tariq Mairaj Rasool Khan

**Affiliations:** 1 Department of Electrical and Computer Engineering, Michigan State University, East Lansing, MI 48824 USA; 2 Department of Electrical Engineering, Bahria University, Khi, 75620 Pakistan; 3 Department of Electrical and Power Engineering, PN Engineering College, National University of Science and Technology, Khi, 75350 Pakistan

**Keywords:** Electrical and electronic engineering, Diagnostic markers

## Abstract

Radiomic features based classifiers and neural networks have shown promising results in tumor classification. The classification performance can be further improved greatly by exploring and incorporating the discriminative features towards cancer into mathematical models. In this research work, we have developed two radiomics driven likelihood models in Computed Tomography(CT) images to classify lung, colon, head and neck cancer. Initially, two diagnostic radiomic signatures were derived by extracting 105 3-D features from 200 lung nodules and by selecting the features with higher average scores from several supervised as well as unsupervised feature ranking algorithms. The signatures obtained from both the ranking approaches were integrated into two mathematical likelihood functions for tumor classification. Validation of the likelihood functions was performed on 265 public data sets of lung, colon, head and neck cancer with high classification rate. The achieved results show robustness of the models and suggest that diagnostic mathematical functions using general tumor phenotype can be successfully developed for cancer diagnosis.

## Introduction

Early diagnosis of cancer can cause timely medical intervention and effective treatment thus preventing progression of the disease from early to advance stages. In such cases, the mortality rate among cancer patients can be significantly reduced. Thus, there is a need of exploring advanced methods for early cancer detection with minimal human intervention.

In recent years, automated cancer diagnostic has emerged as an active area of research. Among several proposed solutions, computational modeling has shown promising results towards cancer diagnosis but these are few to the authors’ best knowledge. Majority of the proposed models have been investigated for lung cancer since lung cancer is one the major causes of death among cancers patients for the last decade^1^. In the work towards quantitative models, Wu *et al*.^2^ presented a likelihood probability model for cancer incidence as a function of age and the number of periodic X-ray screening a male patient has undergone. A multi-factorial likelihood model was proposed by^3^ for MMR gene variant classification of colon cancer based on tumor characteristics and bio-informatics. Beane *et al*.^4^ integrated genomic and clinical features to develop a prediction model for cancer diagnosis. However, these models offer a few limitations such as a small number of potential predictors, generally low overall predictive performance, and methodological constraints.

With all the wealth of knowledge available for the estimation of severity of the disease, the prediction models proposed in the literature are found largely to depend upon the demographics and clinical history of the patient. Recent advances in image acquisition procedures, regularization and image analysis have transformed the quantitative imaging descriptors. These new characteristics could potentially be used as non-invasive diagnostic or predictive biomarkers for cancer. Radiomics is an emerging field of study that uses data mining algorithms to extract quantitative features from the medical images^5^. These quantitative features commonly known as radiomic features provide information about the gray-level patterns and their associations within a region of interest. The radiomic feature analysis has enabled breakthrough to the identification of novel prognostic imaging biomarkers resulting in better understanding of cancer and development of computer aided diagnosis solutions^6,7^. Development of radiomics driven effective mathematical frameworks based on general diagnostic phenotype can further boost the estimation process of cancer diagnosis, just before the symptoms manifest.

In this research study, we have proposed two mathematical likelihood functions for the diagnosis of cancer in CT images. The likelihood functions classify the tumors using the radiomic features with high diagnosis power. Our study showed that it is possible to build a radiomics signature for cancer diagnosis based on general tumor phenotype. The ranking and selection of radiomic features were carried out based on their average scores assigned by 6 supervised and 7 unsupervised feature selection approaches. The training of the proposed classification functions with radiomics integration was performed on 200 lung cancer datasets. The likelihood functions were validated on 165 lung, 35 colon, 30 head and neck malignant tumors and 35 benign lung nodules which shows the robustness of models. The classification results were evaluated in terms of accuracy, sensitivity and specificity. Our presented mathematical models achieved superior tumor classification results when compared with the other state-of-the-art classification algorithms.

The rest of this paper is structured as follows. First, an introduction of the proposed research study, related work and our research contribution are outlined. Then the proposed radiomics based likelihood functions are discussed. Results of the proposed method are followed by a discussion. The research work is summarized with a conclusion.

## Related Work

Radiomic features are quantitative features which are computed to characterize a disease in the medical images. The role of radiomic features in tumor classification has been researched from the broader perspectives of neural networks and machine learning algorithms. Radiomics based classification using machine learning algorithms is a more popular approach and investigates a set of features helpful towards diagnosis followed by the application of classifiers. In this regard, the relationship between radiomic features and the tumor histology was investigated by Wu *et al*.^8^ by applying classifiers of random Forests, naive Bayes, and K-nearest neighbors to the radiomic features. Chen *et al*.^9^ proposed a radiomics signature of four Laws features including minimum, energy, skewness and uniformity and employed Sequential Forward Selection (SFS) and Support Vector Machine (SVM) classifiers for nodule classification. A hierarchical clustering method was used by Choi *et al*.^10^ to identify bounding box anterior–posterior dimension and the standard deviation of inverse difference moment as the top two distinct features for lung cancer diagnosis.

Another progressive approach towards tumor classification is the development of radiomics based efficient neural networks. Liu *et al*.^11^ proposed a multi-view convolutional neural networks (MV-CNN) which used multiple views as input channels, to classify the lung nodules in CT images. Causey *et al*.^12^ proposed a classification neural network based on deep learning features of a lung nodule in CT images. A computer aided diagnosis system was proposed by Kumar *et al*.^13^ which extracted deep features using an auto-encoder coupled with a decision tree classifier to classify the benign and malignant lung nodules.

### Contribution of the proposed work

The proposed research work contributes radiomics based likelihood functions for the diagnosis of cancer in contrast to the previously proposed classification methods in^8–13^ which were motivated by machine learning and neural networks. A mathematical solution incorporating radiomics is investigated to address the tumor classification problem. The proposed computational approach enables accurate and fast classification of a tumor as malignant or benign in CT images and can be further taken up by advance mathematical models to gain in-depth insights of the disease.

To formulate the likelihood functions, diagnostic radiomic signatures were developed which can efficiently detect lung, colon, head and neck cancer. The radiomic signatures were incorporated into mathematical functions which were in turn employed for tumor classification. The performance of radiomic signatures suggest that a radiomic signature can successfully classify a tumor based on the general tumor phenotype.

In addition, the research work has intuitively ranked the 3-D radiomic features of a tumor according to their diagnosis power towards cancer. Two feature ranking lists were prepared using the average score obtained from seven supervised and six unsupervised ranking algorithms. The presented selection approach resulted in accurate feature ranking as it performed feature ranking using multiple ranking algorithms and assigned each algorithm equal weight towards feature selection. In the past studies, feature selection was done by employing any one renowned feature selection algorithm subjecting the ranking potentially to errors^8,10^. This is particularly true since there is no study available in the literature regarding the performance of contemporary feature selection algorithms. Hence, the selection of a feature selection algorithm could affect the features ranks for cancer diagnosis. The assigned rank scores in our study were validated by integrating the two highly ranked features into the proposed likelihood functions for cancer diagnosis.

## Materials and Methods

**Figure 1:**
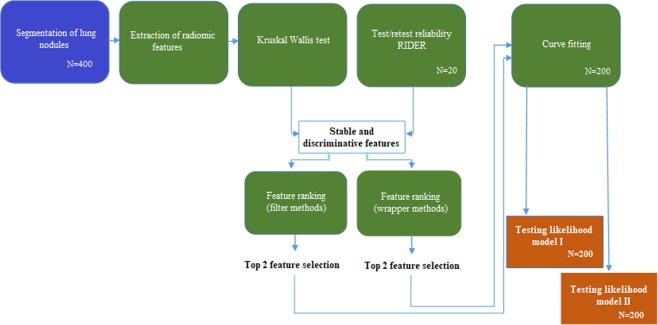
Work flow of the proposed method for nodule classification.

The work flow of the proposed classification functions is shown in Fig. 1. After the data acquisition, tumors segmentation and features extraction; feature selection was performed using two groups of supervised and unsupervised ranking algorithms respectively on the radiomic features of training data sets. Two lists of highly ranked features were obtained from the two selection approaches and the top selected features data were optimally fit into non-linear regression functions.

### Data sets description

The experimental data comprised of 400 lung CT datasets which were accessed from Lung1^14^, LIDC^15^, LUNGx^16^ and RIDER^17^ databases. The other datasets included 35 CT volumes of colon cancer and 30 CT datasets of pre-treatment head and neck cancer (tumor diameter > 10 mm) acquired from CT colongraphy(CTC)^18^ and Head-and-neck squamous cell carcinoma (HNSCC)^19^ databases respectively. Since the largest number of annotated public datasets with benign and malignant tumors are available for lung nodules only, the training cohort was chosen from the lung CT databases. It comprised of 165 malignant and 35 benign lung nodules. The validation cohort included lung nodules, tumors in head, neck and colon. A summary of the employed databases, distribution of the nodule sizes and their types is given in Table 1.

**Table 1:** Distribution summary of employed databases, nodules sizes and their classes.

	Training database (no. of sets)	Validation Database (no. of sets)	Min. Diameter (mm)	Median Diameter (mm)	Max. Diameter (mm)
Malignant Nodules	Lung1(165), RIDER(10)	Lung1(155), CTC(30), HNSCC(35), LIDC(10)	7.015665	66.06505	215.9035
Benign Nodules	LUNGx(35)	LIDC(35)	5.738865	41.58367	221.052

### Tumor segmentation from CT images

The segmentation of lung nodules, polyps in colon and tumors in head and neck were performed using 3-D Slicer platform^20^. The Lung1 database provides the manual segmentation mask for each dataset but the remaining annotated datasets were segmented using the Grow-Cut segmentation algorithm of the platform. The Grow-Cut method is known to perform segmentations which are in high agreement with the manual segmentations^21^.

### Radiomic features extraction

Followed by the segmentation, a total of 105 3-D radiomic features were computed for every tumor. The extracted features belong to 6 feature classes including Shape, Gray level Difference Method (GLDM), First Order Statistics, Gray Level Size Zone Matrix (GLSZM), Gray Level Run Length Matrix (GLRLM) and Neighborhood Gray-Tone Difference Matrix (NGTDM). The number of features selected from each feature class are reported in Table 2. The description of feature classes and complete list of 105 extracted radiomic feature are provided in Supplementary Table S1.

**Table 2:** Description of computed radiomic features.

Features Class	No. of computed features (n = 105)
Shape	13
Gray level Difference Method (GLDM)	14
Gray-Level Co-Occurrence Matrix (GLCM)	23
Neighborhood Gray-Tone Difference Matrix (NGTDM)	5
First order statistics	18
Gray Level Size Zone Matrix (GLSZM)	16
Gray Level Run Length Matrix (GLRLM)	16

### Reliability test and reduction of radiomic features

Prior to the feature selection process, reliability of the computed features was evaluated by carrying out the well- known test of Test-retest reliability. For this purpose, RIDER database has made 20 lung CT datasets available obtained on same-day repeat Computed Tomographic (CT) scans in lung cancer patients. We computed Concordance Correlation Coefficient (CCC) for all the features from repeat scans of RIDER database; and features obtaining a CCC greater than 85% were retained while the rest were excluded. The computed 105 radiomic features were also subjected to Kruskal Wallis test commonly known as One-way ANOVA test to find out the cancer discriminating features for 5% significance level. Based on the results of two tests discussed above, 51 reliable and discriminating features were selected which are listed in Supplementary Material S1.

### Feature ranking algorithms

The finally selected stable and distinct features were ranked according to their diagnosis power towards cancer to further eliminate the redundant features towards classification problem. For this purpose, feature selection algorithms from filter methods and wrapper methods were both considered. The filter methods adopt an unsupervised approach and analyze the inherent distribution properties of the features whereas wrapper methods try to correlate the features properties with class labels. The chosen algorithms under the umbrella of each method are briefly discussed in the following sub-section.

### Radiomic feature ranking using filter methods

A total of seven filter based selection algorithms^22–28^ were chosen based on their high ranking performance reported in the literature for feature ranking. The algorithm in^22^ selects the features exhibiting minimum correlation with each other, whereas the Laplacian score^23^ computes a score for each feature to reflect its locality preserving power. In greedy feature selection technique^24^, a nearest neighbor graph is drawn for all the selected features and the reconstruction error is iteratively computed for the data matrix for the current selected subset to assign ranks. A minimum information loss index for feature ranking is proposed by Mitra *et al*.^25^. Multi-cluster feature selection (MCFS)^26^ technique selects and ranks the features by measuring the correlations between different features by solving the process as a sparse Eigen-problem and a L1-regularized least squares problem. The clustering algorithm^27^ takes into account the relevance of each feature by incorporating it into the framework of Local Learning-Based Clustering (LLC) algorithm. Feature ranking by Zhao *et al*.^28^ is initiated by building a normalized Laplacian matrix from features’ pair-wise similarity graph.

### Radiomic features ranking using wrapper methods

The feature selection process was repeated with the wrapper methods using six well-known ranking algorithms^29–34^. ReliefF Algorithm^29^ penalizes the features that give different values to neighbors of the same binary class, and ranks the features higher that give different values to neighbors of different classes. Feature based Neighborhood Component Analysis (fNCA)^30^ learns feature weights for minimization of an objective function that measures the average leave-one-out regression loss over the training data. Fisher Score^31^ assigns a score to every feature by measuring the ratio of inter-class separation and intra-class variance. The Infinite Latent Feature Selection (ILFS)^32^ algorithm assign ranks to the features by measuring relevancy of all the possible subsets of features using conditional probability. Features Selection via Eigenvector Centrality^33^ ranks the features by mapping the features to a clustering graph and then explores the statistical relationship between pairs of the features. In feature selection with Concave Optimization^34^, the discrimination between two feature classes is made via a separating plane which is obtained by investigating a set of features which could differentiate between the two classes.

### Final feature ranking

**Figure 2:**
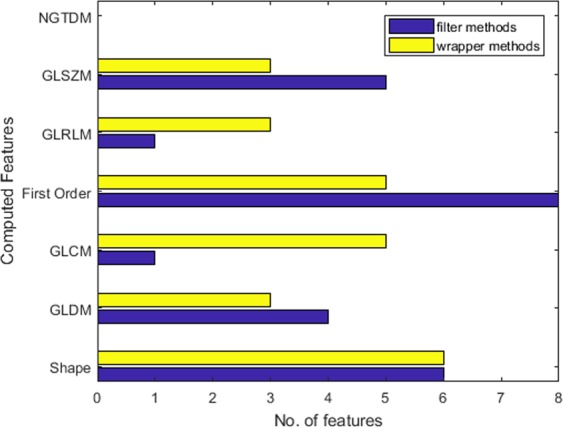
Distribution of top 25 ranked radiomic features with respect to the feature classes.

Using the above-mentioned sets of algorithms, the radiomic features were ranked separately using the wrapper methods and the filter methods. The scores assigned to every feature by each group of ranking algorithms were averaged to obtain the final rank scores of all the features. As mentioned earlier, the purpose of averaging the scores was to assign equal weight to each ranking algorithm in order to obtain accurate feature scores. The average scores of the top 25 selected features computed from the wrapper methods and the filter methods respectively are shown in Fig. S2. In Fig. 2, the distributions of chosen features with respect to their selection method are compared. Evidently, more features from the shape and first order feature classes appear in the top 25 ranking list showing better diagnosis capabilities than the other classes.

The objective of the study was to develop a radiomic signature with two highly discriminative features which are also independent to each other. Such features could be treated as independent variables for the formulation of a likelihood equation. It is noteworthy that more than two features selection did not appear feasible as it could have lead to increased complexity thus reducing the efficiency of the model. We observed that besides the feature classes, computed radiomic features can be broadly categorized in terms of texture and shape. While the shape class describes the shape characteristics of the nodule, the remaining five feature classes compute several properties of the nodule gray levels based on its texture. Since shape and texture offer distinct information about the nodule state, these could be treated independent to each other. Therefore, we chose one feature describing the shape and the other depicting the texture of the nodule as two independent features from the higher ranks of the top 25 ranking list. The selected features are incorporated as independent variables into the proposed likelihood functions.

## Results

The feature extraction in our experiments was carried out using PyRadiomics package^35^, whereas the test-retest reliability and Kruskal Wallis test on the computed features were performed using MATLAB R2018b platform. The feature ranking algorithms and the likelihood functions were also programmed in MATLAB environment. In the following section, we identify the radiomics features for likelihood function formulation followed by performance evaluation of the proposed scheme.

### Development of diagnostic radiomic signatures

Surface Volume ratio(SVR) was the first chosen feature with the highest score in the wrapper based ranking list. It belongs to the shape class and defines the compactness of the nodule. The second selected feature was sum entropy(SE) which is a sum of differences between the neighborhood gray- values. It ranked number 2 on the list, belongs to GLCM class and describes the texture of the nodule. Therefore, the first diagnostic radiomic signature derived from the filter methods ranking comprises of SVR and SE.

The first selected feature from the filter based ranking approach was Large Area Low Gray Level Emphasis(LALGLE) with the highest score on the list. LALGLE belongs to GLSZM feature class and describes the texture of the nodule. It measures the distributions of low intensity based large zones.Volume was the second chosen feature from the shape class with 5^*th*^ rank on the list, since the top 4 features depicted the texture of the nodule. The above selection lead to second diagnostic radiomic signature obtained from the wrapper methods ranking and comprises of LALGLE and volume of the tumor.

### Formulation of radiomics based likelihood functions

The features’ quantitative values in the radiomic signatures were considered as two independent variables *x*_1_ and *x*_2_, then the state (cancer vs. non-cancer) of the nodule for these two features became the dependent variable *y*.

Using 200 training data sets of benign and malignant tumors, the relationship between developed radiomics signatures and the malignancy/benign status of a tumor was quantitatively analyzed and was found to be non-linear. In order to optimally fit a non-linear function to the radiomics data and tumor class, non- linear regression functions^36^ were investigated and the functions fitting the data with minimum possible standard error were finally selected for classification. For this purpose, the above developed two radiomic signatures as two pairs of independent and discriminative features were used to formulate the likelihood models of cancer.

### First mathematical likelihood function (MLF I) using filter methods

The first non-linear regression function fit to the radiomics data using wrapper based selection method is given as follows:

1 $$y=a+bln({x}_{1})+cln({x}_{1}^{2})+\frac{d}{{x}_{2}}+\frac{e}{{x}_{2}^{2}}+\frac{f}{{x}_{2}^{3}}+\frac{g}{{x}_{2}^{4}}$$

Here *x*_1_ denotes the volume value of the test tumor and *x*_2_ describes the Large Area Low Gray Level Emphasis value of the test tumor. The value of *y* is 0 for non-cancer state; and 1 for cancer state of the test nodule. The coefficients of the proposed likelihood function in Eq. (1) are as follows:

$$a=-2.45226185349294;$$ $$b=0.568013700683048;$$ $$c=-2.32311348575522E-02;$$ $$d=-2.68371595182609E-02;$$ $$e=3.61336660703077E-03;$$ $$f=-1.08094045817984E-04;$$ $$g=9.40291849279405E-07$$.

The computed average standard error for the y estimates is 0.30.

### Second mathematical likelihood function (MLF II) using wrapper methods

The following likelihood function is proposed using the radiomic signature from filter based ranking method:

2 $$y=a+b({x}_{1})+c({x}_{1}^{2})+d({x}_{1}^{3})+e({x}_{1}^{4})+fln({x}_{2})$$

Here *x*_1_ denotes the SVR of the test tumor and *x*_2_ denotes the SE value of the test tumor. The value of *y* is 0 for non-cancer state and 1 for cancer state of the test tumor. The coefficients of Eq. (2) assume the following values:

$$a=0.747801694861307$$; $$b=2.22684037581268$$; $$c=-5.58568390095777$$;

$$d=3.631765847909$$; $$e=-0.730551994128231$$; $$f=1.28142101694647E-02$$.

The computed average standard error for the *y* estimate is 0.20. The mathematical functions described by Eq. (1) and Eq. (2) are the proposed radiomics based likelihood functions for cancer diagnosis. These functions can classify a tumor as malignant or benign once the required radiomic features are extracted from CT images and input into their corresponding equations.

### Performance metrics

The performance of the proposed functions for tumor classification is measured in the subsequent sections.

### Lung nodule classification

The likelihood equations, MLF I and MLF II were tested on the radiomic features of 165 malignant nodules and 35 benign nodules from the test cohort. An optimal threshold of $y\ge 0.51$ was chosen to classify a nodule as malignant where as any value of y less than 0.51 classifies the nodule as benign. The diagnosis results of the two models are tabulated in Table 3. Here TP denotes the true positive and is the number of nodules correctly classified malignant whereas FP denotes the false positive and indicates the number of nodules wrongly classified as malignant. FN denotes the false negative and is the number of nodules wrongly interpreted as benign; and TN is the true negative value and denotes the number of nodules correctly classified benign. While MLF I classified 155 malignant and 28 benign nodules correctly, MLF II performed better with correct diagnosis of 161 malignant and 33 benign lung nodules.

**Table 3:** Nodule classification results.

Patient	Test = Positive	Test = Negative
**MLF I Classification**		
Cancer	155(TP)	10(FN)
No Cancer	7(FP)	28(TN)
**MLF II Classification**		
Cancer	161(TP)	4(FN)
No Cancer	2(FP)	33(TN)

The performance of the classification models was quantitatively evaluated with the accuracy, specificity and sensitivity metrics defined as follows:

3 $$Accuracy=\frac{TP+TN}{(TP+FN+FP+TN)}$$

4 $$Specificity=\frac{TP}{(TP+FP)}$$

5 $$Sensitivity=\frac{TN}{(TN+FN)}$$

The first likelihood function MLF I achieved 91.5%(CI:0.864–0.949) accuracy, 95.68%(CI:0.892–0.967) sensitivity and 73.68%(CI:0.579–0.85) specificity in lung nodule classification. Second likelihood function MLF II on the other hand, resulted in an accuracy of 97.0%(CI:0.936–0.989), sensitivity of 98.77%(CI:0.939–0.990) and specificity of 89.19%(CI:0.8139–0.9842) for nodule classification.

**Figure 3:**
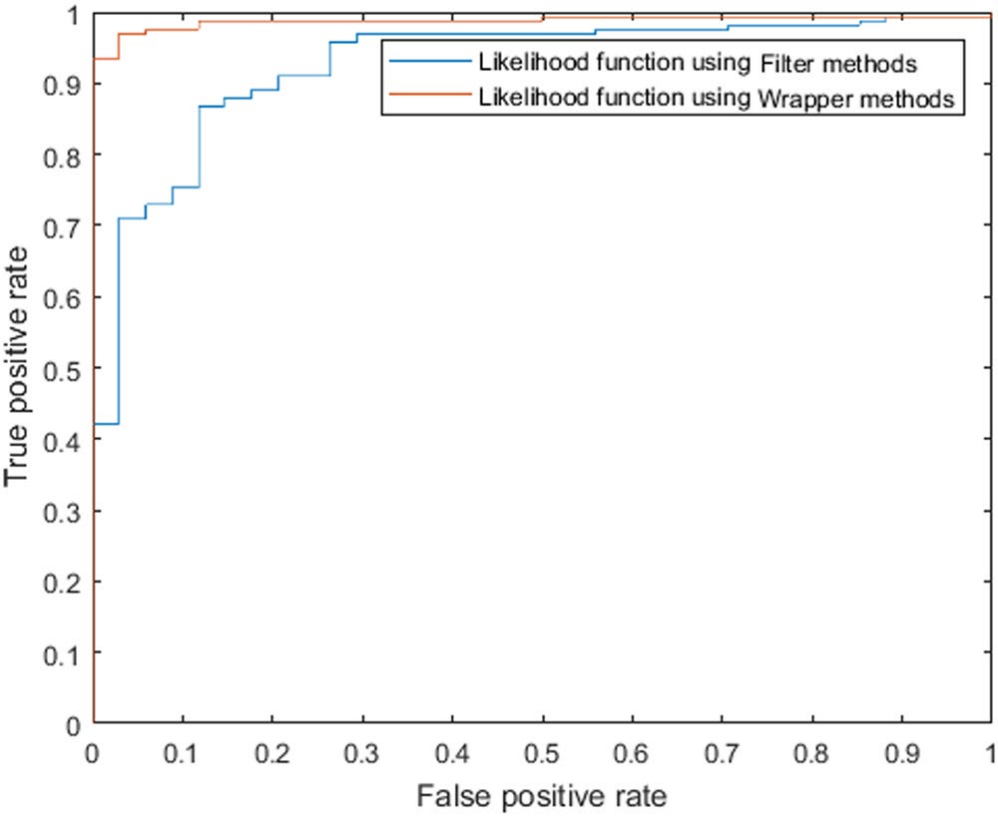
ROC Curves for proposed likelihood functions MLF I and MLF II.

Furthermore, the receiver operating characteristic curves(ROCs) were plotted in Fig. 3 to illustrate the diagnostic ability of two proposed likelihood equations. Higher area under the curves(AUCs) values indicate higher accuracy when two or more methods are compared for various thresholds. The achieved AUCs for MLF I and MLF II were 92.68% and 98.81% respectively which confirm that both the models can discriminate highly between diseased and the non-diseased nodules.

### Malignancy detection in colon, head and neck tumors

A threshold of y = 0.51 and above used in lung nodule classification experiments was also employed for colon, head and neck cancer detection. The likelihood function MLF I detected cancer in 26 out of 35 tumors in colon and 25 out of 30 head-and-neck tumors. The cancer detection rate of MLF I has an accuracy of 74.28% and 83.33% respectively. The second likelihood function MLF II detected cancer in 30 out of 35 colon tumors and 27 out of 30 head and neck tumors correctly with a detection rate of 85.71% and 90% respectively.

### Comparison of MLF I and MLF II

**Figure 4:**
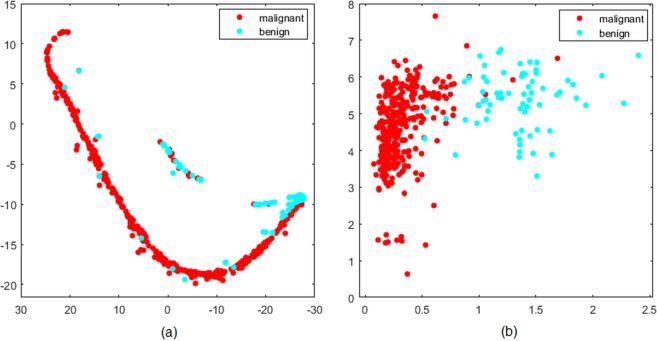
Visualization of (**a**) stable and reliable radiomics features using PCA transformation in image space (**b**) radiomic signature (surface volume ratio, sum entropy) from MLF II in image space.

Although both the likelihood functions have proven to be capable of tumor classification in lung, colon, head and neck; the performance of MLF II was found superior for cancer detection (Acc.% is 97% for lung, 85.71% for colon and 90% for head and neck). The features including surface to volume area and sum entropy in MLF II showed strong ability of cancer diagnosis. The effectiveness of the proposed radiomic signature (surface volume ratio, sum entropy) integrated in MLF II is further demonstrated through their visualization in Fig. 4. Evidently, while the PCA transformed new features failed to differentiate between malignant and benign nodules in Fig. 4(a), surface volume ratio and sum entropy together have successfully identified most of the cancerous and non-cancerous tumors in Fig. 4(b). This comparison further supports the features ranking carried out by the chosen feature selection approach. The achieved results suggest that a diagnostic radiomic signature comprising of one shape and one textural feature can successfully detect multiple types of cancer.

**Figure 5:**
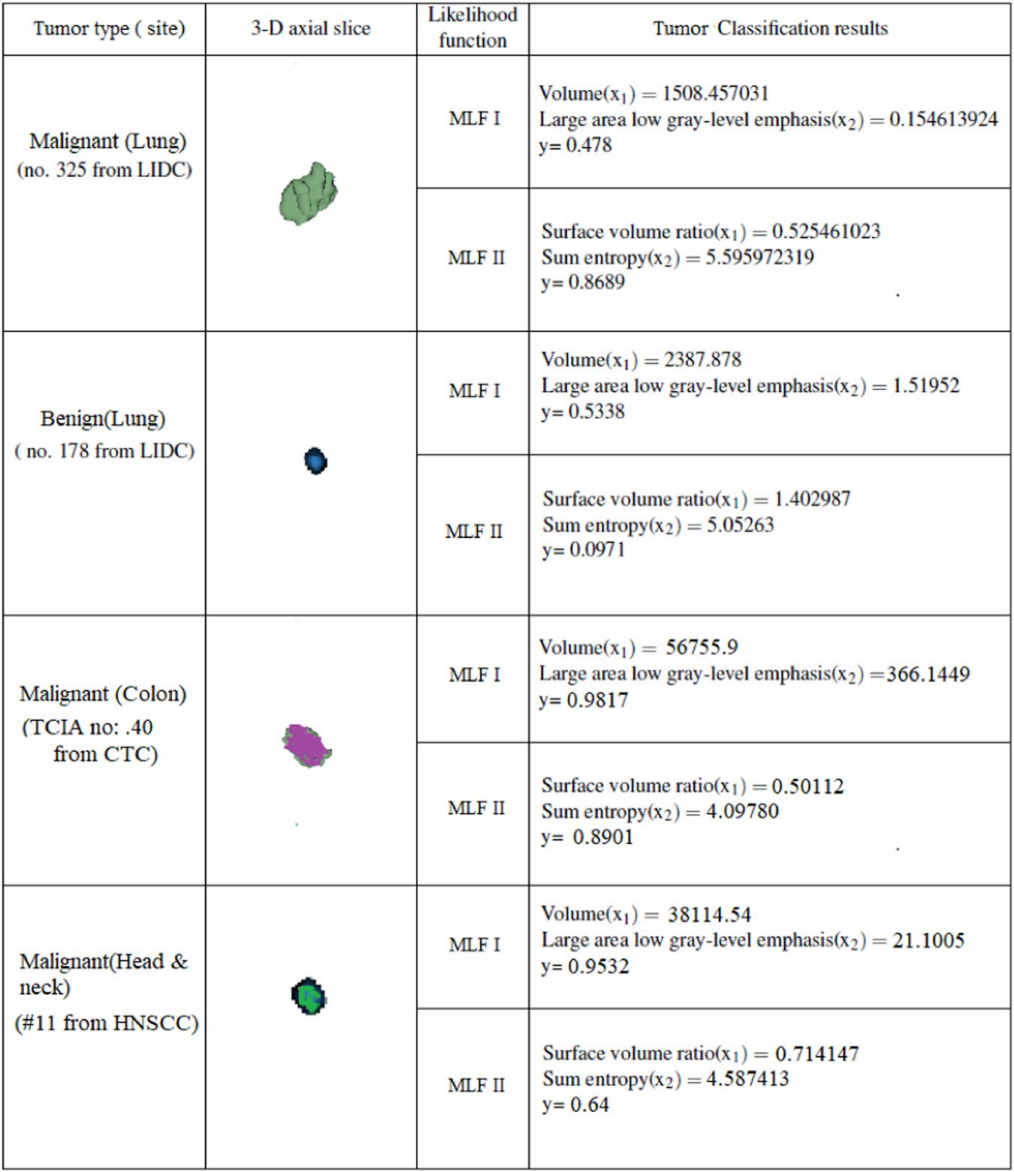
Quantitative classification results of MLF I and II for malignant and benign tumors from LIDC, CTC and HNSCC databases.

It was observed that classifications performed by MLF II(SE = 0.20) were largely correct with values of y obtained either close to 0 or 1 showing excellent classification results. However, MLF I mis-classified quite a few nodules around the chosen cut-off value of y of 0.51. This is most likely due to the comparatively larger standard error(SE = 0.3) contributed by MLF I. The discussed scenario is illustrated in Fig. 5 by reporting the quantitative classification results of both the models for test benign and malignant tumors from LIDC, CTC and HNSCC databases. The obtained values of y in MLF I in the test cases of benign and malignant lung nodules are close to 0.5 and represent wrong diagnosis. On the contrary, the achieved values of y in MLF1 and MLF II for all the other reported cases of lung, colon, head and neck cancer show correct diagnosis and are close to the expected value of 0 or 1.

## Discussion

Radiomics have been an active area of research for medical image analysis and have shown strong correlation with diagnosis and prognosis of cancer. There are still many primary cancer types where the application of radiomics for tumor classification needs in-depth exploration. This includes colon, head and neck cancer as well. Pallamar *et al*. in^37^ have investigated the potential of texture analysis for the differentiation of benign and malignant head and neck tumors in MRI images. The best classification results varied between 81.48%(n = 27) and 92.59%(n = 27) for 1.5 Tesla and 3.0 Tesla acquisition modalities respectively using discriminating features. The results were not encouraging for multi-centre study since tumors classification was poor if benign and malignant tumors were scanned on different sites. The proposed MLF I and MLF II classified head and neck tumors(n = 30) with a detection rate of 83.33% and 90% respectively. The proposed likelihood functions are not only at par with the published results in^37^ but are also robust and independent of acquisition protocols. This is true because the training of the likelihood functions was carried out on datasets acquired from different scanners with varying slice thickness.

Colon cancer is the other cancer where the diagnostic potential of radiomics has remained untapped. Huang *et al*. in^38^ has investigated the gene candidate Notch1 for benign and malignant colon tumors. The Notch 1 expression was expressed in 58% of the colon cancer patients(n = 462). The application of MLF 1 and MLF II for colon cancer detection is the first attempt to employ radiomics for colon cancer diagnosis. The proposed MLF I and MLF II classified colon tumors with a detection rate of 74.28% and 85.71% respectively.

A comparison of the proposed classification models with the other published state-of-the-art classification methods for lung, colon, head and neck tumors is made in Table 4. Since a large number of the research studies on tumor classification for lung cancer are carried out using LIDC database, it is our chosen database as well. The lung cancer classification presented by^11^ reported the highest accuracy of 94.59% (n = 172) using Multi-view convolutional neural networks but the number of benign nodules detected are not mentioned. In the research work presented by^8–10^,^13^, the accuracy, sensitivity and specificity of nodule classification are computed so a full comparison becomes possible. The Random forest classifier in^8^ gave low classification performance with 55% accuracy whereas the validations datasets used by^9,10^,^13^ are small (n = 75, n = 72, n = 97). The quantitative comparison shows that both the likelihood functions MLF I and MLF II have performed better classification than the methods proposed in^8–11^,^13^ of Table 4 with a larger validation set(n = 200). Between the two models, the diagnosis capability of MLF II is proven superior over the other chosen algorithms.

**Table 4:** Comparison of performance metrics of MLF I and MLF II with other state of the art classification models.

	Prediction Model	# of Data sets (Tumor site, Database)	Accuracy	Sensitivity	Specificity	AUC
Wu *et al*.^8^	Random forest classifier	152(Lung, LIDC)	55.0%	80.0%	72.0%	—
Chen *et al*.^9^	SFS, SVM	75(Lung, LIDC)	84.0%	92.85%	72.73%	—
Choi *et al*.^10^	SVM-LASSO	72(Lung, LIDC)	84.6%	87.2%	81.2%	89%
Liu *et al*.^11^	Multi-view convolutional neural networks	172(Lung, LIDC)	94.59%	—	—	98.1%
Kumar *et al*.^13^	Deep convolutional neural network	97(Lung, LIDC)	75.1%	83.35%	61.0%	—
Pallamar *et al*.^37^	Linear Discriminant analysis, k nearest neighbor	27(Head & Neck, Private)	81.48% 1.5T 92.59% 3T	—	—	—
Huang *et al*.^38^	Gene expression	462(Colon, Private)	—	—	—	—
Proposed MLF I	Curve fitting using non-linear regression	200(Lung, LIDC & Lung1) 35(Colon, CTC) 30(Head & Neck, HNSCC)	91.5% 74.28% 83.33%	95.68%	73.68%	92.68%
Proposed MLF II	Curve fitting using non-linear regression	200(Lung, LIDC & Lung1) 35(Colon, CTC) 30(Head & Neck, HNSCC)	97% 85.71% 90%	98.77%	89.19%	98.81%

While CT has been used for lung cancer imaging and CT and MRI both have been used as imaging modalities for head, neck and colon cancer; the proposed models use CT modality only to classify three cancer types with high accuracy. This shows the robustness and benefit of using the proposed likelihood functions over the previously published models.

## Conclusion

In this research work, we have proposed two radiomics based likelihood functions for tumor classification. The research experiments showed that a radiomic signature developed using general tumor phenotype can diagnose multiple cancer types. Intuitive and concise feature selection techniques using wrapper methods and filter methods are presented and compared to distinguish between benign and malignant tumors on CT images. The novelty of our work lies in the radiomics based mathematical approach for tumor classification problem for colon, lung, head and neck which has the potential to classify several other cancer types. The proposed classification functions are easy to implement and have demonstrated better performance in terms of accuracy, sensitivity and specificity when compared with the other existing competent techniques. We believe that the presented study opens a new research avenue in the domain of mathematical and stochastic modelling and has strong potential for further exploration in cancer diagnostics.

## Supplementary information

Supplementary material

## Data Availability

The datasets can be accessed from the following URLs for reproducibility purpose: LIDC, Lung1, RIDER, LUNGx, CT Colonography, Head and neck cancer, The MATLAB code used to carry out the several tasks in the research study can be accessed at: MATLAB Code.
