# Supplementary material for: Radiomics based likelihood functions for cancer diagnosis
Source: Sci Rep. 2019 Jul 1;9:9501. doi: 10.1038/s41598-019-45053-x (PMC6603029; doi:10.1038/s41598-019-45053-x)
Supplement: Supplementary file 1 — Supplementary material [file 41598_2019_45053_MOESM1_ESM.docx]

**Title: Radiomics based likelihood functions for cancer diagnosis**

**Authors:** Hina Shakir, Yiming Deng, Haroon Rasheed and Tariq Mairaj Rasool Khan

**TABLE S1: List of 3-D features extracted from the nodules of experimental CT datasets**

| **Feature Class(Description)** | **Computed features** |
| --- | --- |
| Shape(Describes shape) | 1. 1.Maximum 3D Diameter 2. Maximum 2D Diameter Slice 3. Sphericity 4. Minor Axis 5. Elongation 6. Surface Volume Ratio 7. Volume 8. Major Axis 9. Surface Area 10. Flatness 11. Least Axis 12. Maximum 2D Diameter Column 13. Maximum 2D Diameter Row |
| Gray level Difference Method(GLDM)( Extracts the statistical texture Features) | 1. Gray Level Variance 2. High Gray Level Emphasis 3. Dependence Entropy 4. Dependence Non Uniformity 5. Gray Level Non Uniformity 6. Small Dependence Emphasis 7. Small Dependence High Gray Level Emphasis 8. Dependence Non Uniformity Normalized 9. Large Dependence Emphasis 10. Large Dependence Low Gray Level Emphasis 11. Dependence Variance 12. Large Dependence High Gray Level Emphasis 13. Small Dependence Low Gray Level Emphasis 14. Low Gray Level Emphasis |
| Gray-Level Co-Occurrence Matrix (GLCM)( Describes texture by means of  second order statistics) | 1. Joint Average 2. Sum Average 3. Joint Entropy 4. Cluster Shade 5. Maximum Probability 6. Idmn 7. Joint Energy 8. Contrast 9. Difference Entropy 10. Inverse Variance 11. Difference Variance 12. Idn 13. Idm 14. Correlation 15. Autocorrelation 16. Sum Entropy 17. Sum Squares 18. Cluster Prominence 19. Imc2 20. Imc1 21. Difference Average 22. Id 23. Cluster Tendency |
| First Order (Measures the voxels gray-levels  spread within a region of interest using histograms) | 1. Interquartile Range 2. Skewness 3. Uniformity 4. Median 5. Energy 6. Robust Mean Absolute Deviation 7. Mean Absolute Deviation 8. Total Energy 9. Maximum 10. Root Mean Squared 11. 90 Percentile 12. Minimum 13. Entropy 14. Range 15. Variance 16. 10 Percentile 17. Kurtosis 18. Mean 19. Standard Deviation |
| Gray Level Run Length Matrix( GLRLM)( Computes the continuous run of pixels of the similar gray - values, in a particular direction) | 1. Short Run Low Gray Level Emphasis 2. Gray Level Variance 3. Low Gray Level Run Emphasis 4. Gray Level Non Uniformity Normalized 5. Run Variance 6. Gray Level Non Uniformity 7. Long Run Emphasis 8. Short Run High Gray Level Emphasis 9. Run Length Non Uniformity 10. Short Run Emphasis 11. Long Run High Gray Level Emphasis 12. Run Percentage 13. Long Run Low Gray Level Emphasis 14. Run Entropy 15. High Gray Level Run Emphasis 16. Run Length Non Uniformity Normalized |
| Gray Level Size Zone Matrix (GLSZM)( Computes the number of linked voxels with similar gray level intensity) | 1. Gray Level Variance 2. Zone Variance 3. Gray Level Non Uniformity Normalized 4. Size Zone Non Uniformity Normalized 5. Size Zone Non Uniformity 6. Gray Level Non Uniformity 7. Large Area Emphasis 8. Small Area High Gray Level Emphasis 9. Zone Percentage 10. Large Area Low Gray Level Emphasis 11. Large Area High Gray Level Emphasis 12. High Gray Level Zone Emphasis 13. Small Area Emphasis 14. Low Gray Level Zone Emphasis 15. Zone Entropy 16. Small Area Low Gray Level Emphasis |
| Neighborhood Gray-Tone Difference Matrix NGTDM(Computes the difference of intensity of a pixel with its neighboring pixels) | 1. Coarseness 2. Complexity 3. Strength 4. Contrast |

**SUPPLEMENTARY MATERIAL S I**

The reliable and stable features selected after test-retest reliability and Kruskal Wallis test belonged to 5 feature classes and no feature was selected from Neighborhood Gray-Tone Difference Matrix (NGTDM**).** Following is the list of 51 selected 3 –D radiomics features which were subjected to the feature ranking algorithms for feature selection:

**Shape Class**


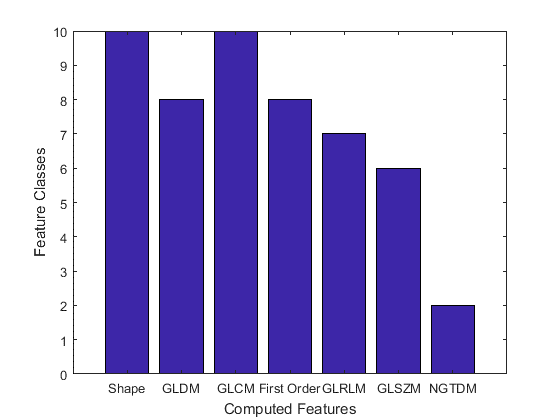


| 1. Maximum3DDiameter |
| --- |
| 1. Maximum2DDiameterSlice |
| 1. Minor Axis |
| 1. Surface Volume Ratio |
| 1. Volume |
| 1. Major Axis |
| 1. Surface Area |
| 1. Least Axis |
| 1. Maximum2DDiameterColumn |
| 1. Maximum2DDiameterRow |

**Gray level Difference Method (GLDM)**

Figure S1

| 1. High Gray Level Emphasis |
| --- |
| 1. Gray Level Non Uniformity |
| 1. Small Dependence High Gray Level Emphasis |
| 1. Dependence Non Uniformity Normalized |
| 1. Dependence Variance |
| 1. Large Dependence High Gray Level Emphasis |
| 1. Small Dependence Low Gray Level Emphasis |
| 1. Low Gray Level Emphasis |

**Gray-Level Co-Occurrence Matrix (GLCM)**

| 1. Joint Entropy |
| --- |
| 1. Maximum Probability |
| 1. Joint Energy |
| 1. Difference Entropy |
| 1. Inverse Variance |
| 1. Difference Variance |
| 1. Sum Entropy |
| 1. Sum Squares |
| 1. Cluster Prominence |
| 1. Difference Average |

**Gray Level Size Zone Matrix (GLSZM)**

| 1. Zone Variance |
| --- |
| 1. Gray Level Non Uniformity Normalized |
| 1. Large Area Emphasis |
| 1. Zone Percentage |
| 1. Large Area Low Gray Level Emphasis |
| 1. Large Area High Gray Level Emphasis |

**Gray Level Run Length Matrix (GLRLM)**

| 1. Short Run Low Gray Level Emphasis |
| --- |
| 1. Low Gray Level Run Emphasis |
| 1. Gray Level Non Uniformity |
| 1. Run Percentage |
| 1. Long Run Low Gray Level Emphasis |
| 1. Run Entropy |
| 1. Run Length Non Uniformity Normalized |

**Neighborhood Gray-Tone Difference Matrix (NGTDM)**

| 1. Coarseness |
| --- |
| 1. Busyness |

**First Order**

| 1. Interquartile Range |
| --- |
| 1. Uniformity |
| 1. Mean Absolute Deviation |
| 1. Maximum |
| 1. 90 Percentile |
| 1. Entropy |
| 1. Variance |
| 1. Kurtosis |

The distribution of selected features with respect to the feature class is shown in Figure S1. The distribution of top 25 ranked radiomic features (a) using filter methods (b) using wrapper methods is shown in Figure S2.


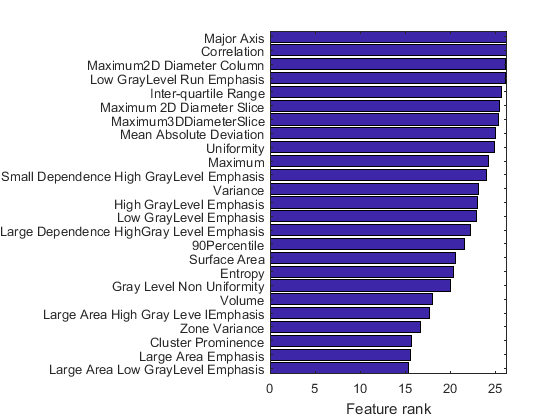


(a)
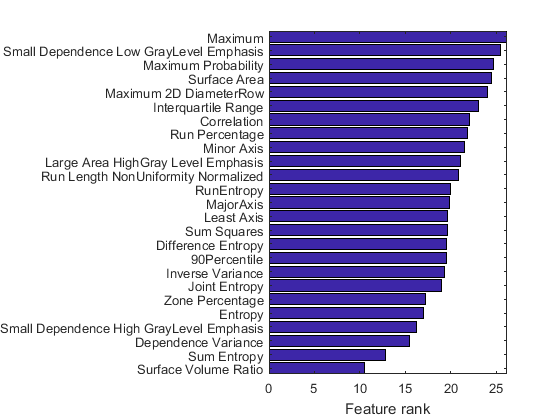


(b)

Figure S2: Top 25 ranked radiomic features using(a) filter methods (b)wrapper methods
